# Fruquintinib versus placebo in patients with refractory metastatic colorectal cancer: safety analysis of FRESCO-2

**DOI:** 10.1093/oncolo/oyae360

**Published:** 2025-03-31

**Authors:** Cathy Eng, Arvind Dasari, Sara Lonardi, Rocio Garcia-Carbonero, Elena Elez, Takayuki Yoshino, Alberto Sobrero, James Yao, Pilar Garcia-Alfonso, Judit Kocsis, Antonio Cubillo Gracian, Andrea Sartore-Bianchi, Taroh Satoh, Violaine Randrian, Jiri Tomasek, Geoff Chong, Zhao Yang, Ferdinand Guevara, William Schelman, Rajash Pallai, Josep Tabernero

**Affiliations:** Department of Medicine, Division of Hematology and Oncology, Vanderbilt Ingram Cancer Center, Nashville, TN 37232, United States; Department of Gastrointestinal Medical Oncology, The University of Texas MD Anderson Cancer Center, Houston, TX 77030, United States; Medical Oncology Unit 1, Veneto Institute of Oncology IOV-IRCCS Padua, Padua 35128, Italy; Oncology Department, Hospital Universitario 12 de Octubre, Facultad de Medicina, Universidad Complutense de Madrid, Madrid 28041, Spain; Vall d’Hebron Barcelona Hospital Campus, Vall d’Hebron Institute of Oncology (VHIO), Barcelona 08035, Spain; Department of Gastroenterology and Gastrointestinal Oncology, National Cancer Center Hospital East, Kashiwa, Chiba 277-8577, Japan; Department of Medical Oncology, Azienda Ospedaliera San Martino, Genoa 16132, Italy; Department of Gastrointestinal Medical Oncology, The University of Texas MD Anderson Cancer Center, Houston, TX 77030, United States; Medical Oncology, Hospital Universitario Gregorio Marañón, Madrid 28007, Spain; Department of Oncoradiology, Bács-Kiskun Megyei Oktatókórház, Kecskemét 6000, Hungary; Medical Oncology, HM Universitario Sanchinarro Centro Integral Oncológico Clara Campal, Madrid 28050, Spain; Department of Oncology and Hemato-Oncology, Università degli Studi di Milano, Milan 20122, Italy; Department of Gastroenterological Surgery, Graduate School of Medicine, Osaka University, Suita 565-0871, Japan; Hepato-Gastroenterology Department, CHU de Poitiers, F-86000 Poitiers, France; Department of Complex Oncology Care, Masaryk Memorial Cancer Institute, Brno 60200, Czech Republic; Olivia Newton John Cancer & Wellness Centre, Austin Hospital, Heidelberg, VIC 3084, Australia; HUTCHMED International Corporation., Florham Park, NJ 07932, United States; HUTCHMED International Corporation., Florham Park, NJ 07932, United States; HUTCHMED International Corporation., Florham Park, NJ 07932, United States; HUTCHMED International Corporation., Florham Park, NJ 07932, United States; Vall d’Hebron Barcelona Hospital Campus, Vall d’Hebron Institute of Oncology (VHIO), Barcelona 08035, Spain

**Keywords:** metastatic colorectal cancer, adverse event management, phase 3, placebo-controlled, adverse events of special interest, oral

## Abstract

**Background:**

Fruquintinib is a highly selective, oral inhibitor of all 3 VEGF receptors. The global, randomized, double-blind phase 3 FRESCO-2 trial (NCT04322539) met its primary endpoint demonstrating significantly improved overall survival in patients with refractory metastatic colorectal cancer (mCRC) who received fruquintinib plus best supportive care (BSC) versus placebo plus BSC. Here we report detailed safety data from FRESCO-2 including an analysis of treatment-related adverse events of special interest (AESIs).

**Patients and methods:**

Patients with mCRC eligible for FRESCO-2 had received all standard chemotherapies and prior anti-VEGF and anti-EGFR therapies if indicated, and displayed progression on, or intolerance to, TAS-102 and/or regorafenib. Prespecified AESIs based on VEGFR tyrosine kinase inhibitor drug classes were evaluated.

**Results:**

Incidences of treatment-related AESIs were 64.9% with fruquintinib + BSC versus 23.0% with placebo + BSC. The most frequent all-grade treatment-related AESIs for fruquintinib were hypertension (28.9%; grade ≥3 10.7%), palmar-plantar erythrodysesthesia syndrome/hand-foot skin reaction (PPE 18.6%; grade ≥3 6.1%), and hypothyroidism (15.6%; grade ≥3 0.4%). Dose reductions due to treatment-related AESIs were reported in 10.3% of patients who received fruquintinib + BSC versus 0.4% with placebo + BSC. The most common treatment-related AESIs resulting in dose reduction for fruquintinib were PPE syndrome (5.0%), hypertension (2.9%), and proteinuria (1.3%). Overall, 5.9% versus 0.9% had treatment-related AESIs resulting in study drug discontinuation.

**Conclusion:**

Fruquintinib + BSC demonstrated a predictable and manageable safety profile in pretreated patients with mCRC and is a novel oral treatment option that prolongs survival and enriches the continuum of care in this population.

## Graphical Abstract

**Graphical Abstract:**
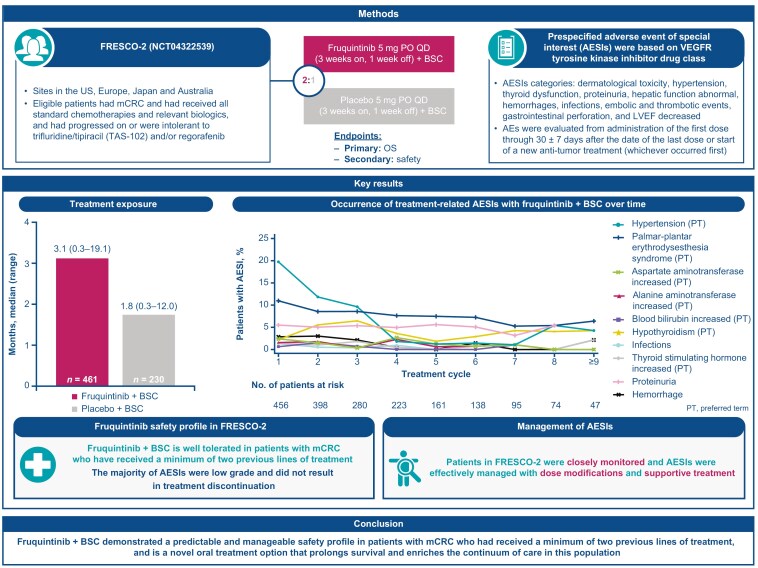

**Implications for practice**

Fruquintinib is a highly selective oral inhibitor of all 3 VEGF receptors, approved for patients with previously treated metastatic colorectal cancer (mCRC), regardless of biomarker status. In the phase 3 FRESCO-2 study, the most frequent any grade treatment-related adverse events of special interest (AESIs) reported with fruquintinib were hypertension, palmar-plantar erythrodysesthesia syndrome, and hypothyroidism. These AESIs were expected based on the mechanism of action; the majority were low grade, manageable, and did not result in discontinuation. Fruquintinib is a novel oral treatment option for patients with mCRC that is well tolerated, prolongs survival, and enriches the continuum of care.

## Introduction

Globally, colorectal cancer (CRC) is the third most diagnosed cancer and the second leading cause of cancer-related mortality.^1^ In 2023, it was estimated that 7.8% of new cancer cases and 8.6% of deaths worldwide were attributed to CRC.^2^ Metastatic disease is common in CRC with up to 50% of patients with localized disease developing metastases.^3^ The prognosis for metastatic CRC (mCRC) remains unsatisfactory with a 5-year relative survival of ~14% for distant disease.^2,4^

Numerous treatment options are available for mCRC including chemotherapy and biologics targeting the vascular endothelial growth factor (VEGF), epidermal growth factor receptor (EGFR), and human epidermal growth factor receptor 2 pathways, BRAF V600E mutations, and the mismatch repair deficient (dMMR)/microsatellite instability-high (MSI-high) population.^5-10^ The majority of patients with mCRC do not have driver genomic alterations that can be treated with targeted therapy.^11,12^ For these patients, and those whose disease has progressed despite having received standard treatments, approved treatment options are limited to the oral cytotoxic chemotherapeutic trifluridine/tipiracil (TAS-102) and the multi-kinase inhibitor regorafenib. Both these agents have reported improvements of less than 2 months in median overall survival (OS) compared with placebo plus best supportive care (BSC) in pivotal clinical trials, in which a majority of patients had received 2 or more previous lines of treatment.^13,14^ However, the addition of bevacizumab to TAS-102 in a recent study increased the median OS by more than 2 months compared to single agent TAS-102.^15^ Additionally, TAS-102 and regorafenib may be associated with unmanageable and serious toxicities, such as myelosuppression for TAS-102,^16,17^ and hepatotoxicity for regorafenib.^18,19^ For pretreated patients with mCRC who have progressed on or are intolerant to these therapies, well-tolerated and effective options are limited.^20^

Fruquintinib is a highly selective, oral inhibitor of all 3 VEGF receptors (VEGFR-1, -2, and -3), which restricts tumor growth and progression through inhibition of angiogenesis.^21^ Preclinical and phase I analyses showed that the enhanced target selectivity of fruquintinib limited off-target kinase activity with weak to no inhibitory effects of fruquintinib on all other kinases.^20-22^ Based on the phase III FRESCO study (NCT02314819), fruquintinib was approved in China for patients with mCRC who have received a minimum of 2 prior systemic antineoplastic therapies,^23^ including fluoropyrimidine, oxaliplatin, and irinotecan, with or without prior use of anti-VEGF or anti-EGFR therapies.^20^ FRESCO met its primary endpoint demonstrating a significant absolute improvement in OS of 2.7 months with fruquintinib plus BSC versus placebo plus BSC (median OS, 9.3 vs 6.6 months; hazard ratio [HR]: 0.65; *P* < .001).^24^

At the time of FRESCO, treatment patterns for mCRC in China differed from those in the rest of the world. Therefore, among patients included in FRESCO, few had received prior VEGF inhibitors (30%) or EGFR antibodies (14%); none had received TAS-102, and those exposed to regorafenib were excluded.^24^ Patients in FRESCO had to have received a minimum of 2 prior lines of treatment and 21% had received more than 3 prior lines. FRESCO-2 was designed to investigate fruquintinib in a population that better reflected global treatment practice patterns by ensuring that patients treated with regorafenib and/or TAS-102 were not excluded.^20^ Thus, most patients in FRESCO-2 had received more than 3 prior lines of treatment for metastatic disease (73%).^25^

In FRESCO-2, fruquintinib plus BSC demonstrated statistically significant and clinically meaningful improvements in OS (median 7.4 vs 4.8 months; HR: 0.66; *P* < .001) and progression-free survival (PFS, median 3.7 vs 1.8 months; HR: 0.32; *P* < .001) versus placebo plus BSC^25^ as well as a favorable safety profile, along with no deterioration in the quality of life.^26^ Based on the results of FRESCO and FRESCO-2, fruquintinib was approved by the US Food and Drug Administration for the treatment of adult patients with mCRC who have been previously treated with fluoropyrimidine-, oxaliplatin-, and irinotecan-based chemotherapy, anti-VEGF therapy, and, if *RAS* wild-type and medically appropriate, anti-EGFR therapy.^27^ Here we report detailed safety data from FRESCO-2 including analysis of treatment-related adverse events of special interest (AESIs) and guidance on how to manage them clinically.

## Patients and methods

The design of FRESCO-2 has been described previously including eligibility criteria for enrollment in full.^20,25^ Briefly, patients aged ≥18 years (≥20 years in Japan) with histologically and/or cytologically documented mCRC were eligible if they had received all standard treatments including fluoropyrimidine-, oxaliplatin-, and irinotecan-based chemotherapy, anti-VEGF therapy, and, if *RAS* wild type, anti-EGFR therapy and had progressed on, or been intolerant of, TAS-102 and/or regorafenib. Furthermore, patients with MSI-high or dMMR tumors or *BRAFV600E*-mutant tumors must have also received an immune checkpoint inhibitor or BRAF inhibitor, respectively. Additional inclusion criteria included an Eastern Cooperative Oncology Group performance status (ECOG PS) score of 0-1. Patients were excluded if they had inadequate organ function, characterized by serum total bilirubin >1.5 upper limit of normal (ULN), alanine aminotransferase (ALT) or aspartate aminotransferase (AST) >2.5/>5 ULN without/with hepatic metastases, serum creatinine >1.5 ULN, or creatinine clearance <60 mL/min, protein urinalysis ≥2+ or 24-hour urine protein ≥1.0 g/24-hour, systolic blood pressure ≥140 mmHg and/or diastolic blood pressure ≥90 mmHg, International Normalized Ratio >1.5 ULN or activated partial thromboplastin time >1.5 ULN, unless the patient was currently receiving or intended to receive a prophylactic anticoagulant. Additional exclusion criteria included a history of a thromboembolic event, including deep vein thrombosis, pulmonary embolism, or arterial embolism within 6 months prior to screening.

Eligible patients were randomized in a 2:1 ratio to receive fruquintinib 5 mg or matching placebo given orally once a day (QD) on days 1-21 in a 28-day cycle plus BSC determined by local clinical practices and guidelines. Randomization was stratified by previous therapy (TAS-102 or regorafenib, or both), *RAS* mutation status (wild-type or mutant), and duration of metastatic disease (≤ or >18 months)**.** To prevent unintentional enrichment, the number of patients previously treated with regorafenib was limited to 50% of the total randomized patients**.** Patients received treatment until disease progression, death, unacceptable toxicity, withdrawal of consent, discontinuation based on physician decision, or study completion or termination.

The primary objective was to evaluate the OS of fruquintinib plus BSC compared to placebo plus BSC. Safety evaluation was a secondary objective and was conducted in all patients who received at least one dose of fruquintinib or placebo (safety population). Safety endpoints included adverse events (AEs); the type, incidence, and intensity of treatment-emergent AEs were evaluated from administration of the first dose of fruquintinib or placebo through 30 ± 7 days after the date of the last dose or start of a new anti-tumor treatment (whichever occurred first). Medical history and all AEs were coded using the Medical Dictionary for Regulatory Activities (MedDRA version 25.0) and were graded according to the National Cancer Institute Common Terminology Criteria for Adverse Events, version 5.0. The seriousness of AEs and the relationship to study treatment were determined by the investigator. Dose modifications were permitted, and guidance was provided for risks identified from previous clinical and non-clinical studies, including dermatological toxicities, hypertension, proteinuria, decreased platelet count, hemorrhage, and abnormal liver function (Supplementary Table S1). Hypertension was closely monitored and effectively managed with antihypertensives selected according to the clinical characteristics of the individual patients and following antihypertensive treatment guidelines such as the American Heart Association/American College of Cardiology hypertension treatment guidelines.^28^ Grade 3 hypertension was additionally managed with dose holds, and if resolved or recovered to grade 1, treatment was resumed at the next lower dose. Fruquintinib was discontinued for grade 4 hypertension events. For dermatological toxicities, such as palmar-plantar erythrodysesthesia (PPE) syndrome, active supportive treatments such as moisturizing skin cream, lotion, or hydrophilic urea ointment were recommended to relieve the symptoms; with the fruquintinib dose being held for any grade 2 or 3 events with treatment resumed upon recovery (at the same dose if it was grade 2 or next lower dose if it was grade 3). Proteinuria was closely monitored during screening and throughout treatment. For high grade 2 proteinuria (≥ 2 g/24 hour) the fruquintinib dose was held with treatment resumed at the next lower dose upon recovery or resolution to <1 g/24 hour; fruquintinib was permanently discontinued for nephrotic syndrome or if proteinuria did not recover to <1g/24 hour. There were no specific dose reduction or management guidelines required for hypothyroidism; grade 3 events were managed with hormone replacement and dose holds until resolution or recovery to grade 1 and treatment resumed at the next lower dose; fruquintinib was discontinued for grade 4 events. Overall, patients were allowed to have no more than 2 dose reductions: one from 5 mg QD to 4 mg QD and, if not tolerated, then a second reduction from 4 mg QD to 3 mg QD. Once a dose had been reduced, it could not be re-escalated. Prophylactic use of anticoagulants, antiemetics, granulocyte colony stimulating factors, granulocyte macrophage colony-stimulating factors, platelet stimulating factors, or erythropoietin was permitted as clinically indicated.

This report focuses on prespecified AESIs based on VEGFR tyrosine kinase inhibitor drug classes. AESIs were classified into 10 categories according to MedDRA terms and were further filtered (narrowed and/or broadened depending on the category) by the respective standardized MedDRA Queries (SMQ) search criteria. The selected AESIs categories assessed were dermatological toxicity (defined by the MedDRA system organ class [SOC] “skin and subcutaneous tissue disorders”), hypertension (defined by the MedDRA SMQ “hypertension” [narrow]), thyroid dysfunction (defined by the MedDRA SMQ “thyroid dysfunction” [broad]), proteinuria (defined by the MedDRA SMQ “proteinuria” [narrow]), hepatic function abnormal (defined by the MedDRA SMQ “drug-related hepatic disorder-comprehensive search” [narrow]), hemorrhages (defined by the MedDRA SMQ “hemorrhages” [narrow]), infections (defined by the MedDRA SOC “infections and infestations” [narrow]), embolic and thrombotic events (defined by the MedDRA SMQ “embolic and thrombotic event” [narrow]), gastrointestinal perforation (defined by the MedDRA SMQ “gastrointestinal perforation” [narrow]), and left ventricular ejection fraction decreased (defined by the MedDRA SMQ “cardiac failure” [narrow]). AESIs were evaluated for frequency, time to first onset, and occurrence over time. All safety outcomes are presented using descriptive statistics using SAS, version 9.4 (SAS Institute Inc.). Within these AESI categories, the following preferred terms were assessed: hypertension, PPE syndrome, also called hand-foot skin reaction, AST increased, ALT increased, blood bilirubin increased, hypothyroidism, and thyroid stimulating hormone increased.

The study was conducted in accordance with the Declaration of Helsinki and Good Clinical Practices, including the International Council for Harmonization. The protocol and amendments were approved by the institutional review board and independent ethics committee at each participating site. All patients provided written informed consent at enrollment.

## Results

### Patient characteristics and primary endpoint

In the FRESCO-2 study, 691 patients were randomized to receive fruquintinib plus BSC (*n* = 461) or placebo plus BSC (*n* = 230) between August 12, 2020 and December 2, 2021. FRESCO-2 met its primary endpoint, with patients who received fruquintinib plus BSC demonstrating a clinically meaningful improvement in OS compared with those who received placebo plus BSC (median 7.4 vs 4.8 months; HR: 0.66; *P* < .001).^25^ OS rates at 6 months were 60.4% (95% CI, 55.9-64.9) in patients receiving fruquintinib versus 41.5% (95% CI, 35.0-48.0) in patients receiving placebo, and OS rates at 9 months were 41.1% (95% CI, 36.4-45.8) in patients receiving fruquintinib versus 28.2% (95% CI, 22.1-34.3), in patients receiving placebo. PFS was also improved with fruquintinib versus placebo with 6-month PFS rates of 23.8% (95% CI, 19.7-28.0) versus 1.1% (95% CI, 0-2.6) and 9-month PFS rates of 11.3% (95% CI, 8.1-14.6) versus 0.5% (95% CI, 0-1.6).

The safety population comprised 686 patients: 3 patients randomized to receive fruquintinib did not receive treatment and 2 patients received placebo instead (fruquintinib plus BSC: *n* = 456); 2 patients randomized to placebo did not receive treatment (placebo plus BSC: *n* = 230). The baseline demographics and disease characteristics were well-balanced between the 2 treatment arms (**Table 1**). The median age in both groups was 64 years and the majority of patients had an ECOG PS of 1. In the fruquintinib plus BSC group versus the placebo plus BSC group, the most frequently reported relevant medical history at study entry included hypertension (preferred term; 49.8% vs 52.2%), infections and infestations (SOC, 14.3% vs 16.5%; including the preferred term urinary tract infection), and investigations (SOC, 10.1% vs 10.4%; including the preferred terms increased AST, ALT, and blood bilirubin) (Supplementary Table S2).

**Table 1.** Patient demographics and diseases characteristics at baseline (safety population).

Characteristics, *n* (%)^*^	Fruquintinib + BSC (*n* = 456)	Placebo + BSC (*n* = 230)
Age, years		
Median (range)	64 (56-70)	64 (57-69)
≥65	212 (46.5)	112 (48.7)
Sex		
Female	215 (47.1)	90 (39.1)
Male	241 (52.9)	140 (60.9)
ECOG PS		
0	193 (42.3)	102 (44.3)
1	263 (57.7)	128 (55.7)
Liver metastases	335 (73.5)	155 (67.4)
Prior therapies		
Anti-VEGF	440 (96.5)	221 (96.1)
Anti-EGFR	179 (39.3)	88 (38.3)
Prior TAS-102 and/or regorafenib		
TAS-102	237 (52.0)	121 (52.6)
Regorafenib	40 (8.8)	18 (7.8)
Both	179 (39.3)	91 (39.6)
Number of prior treatment lines in metastatic disease		
Median (range)	4 (2-16)	4 (2-12)
≤3	124 (27.2)	64 (27.8)
>3	332 (72.8)	166 (72.2)
*RAS* status		
Wild type	169 (37.1)	85 (37.0)
Mutant	287 (62.9)	145 (63.0)
*BRAF* status		
Wild type	397 (87.1)	197 (85.7)
V600E mutation	7 (1.5)	10 (4.3)
Other	52 (11.4)	23 (10.0)
Microsatellite or mismatch repair status		
MSS and/or pMMR	422 (92.5)	215 (93.5)
MSI-H and/or dMMR	5 (1.1)	4 (1.7)
Unknown	29 (6.4)	11 (4.8)

^*^Percentages are based on the number of patients in each treatment group unless otherwise stated.

Abbreviations: BSC, best supportive care; dMMR, deficient mismatch repair; ECOG PS, Eastern Cooperative Oncology Group performance status; EGFR, epidermal growth factor receptor; MSI-H, high microsatellite instability; MSS, microsatellite stable; pMMR, proficient mismatch repair; TAS, trifluridine/tipiracil; VEGF, vascular endothelial growth factor.

### Treatment exposure

Patients in the fruquintinib group had longer treatment exposure (median 3.06 months, range, 0.3-19.1) than in the placebo group (median 1.84 months, range, 0.3-12.0). The daily target dose of fruquintinib was 3.75 mg (total cycle dose [5 mg × 21 days] divided by cycle length [28 days]) and median daily fruquintinib dose was maintained until cycle 5, and gradually decreasing thereafter (Supplementary Figure S1). The median relative dose intensity was 91.6% with fruquintinib versus 97.6% with placebo.

### Safety and tolerability

The overall safety profile of fruquintinib has been previously reported^25,29^; treatment-related any-grade and grade ≥3 AEs reported with fruquintinib plus BSC and placebo plus BSC are shown in Supplementary Table S3; the most frequently reported (>15% of patients) any-grade treatment-related AEs with fruquintinib were hypertension (28.9%), asthenia (24.6%), PPE syndrome (18.6%), diarrhea (18.0%), and decreased appetite (16.0%). In the fruquintinib plus BSC and placebo plus BSC groups, 64.9% and 23.0% of patients, respectively, reported any treatment-related AESI; the most frequently reported treatment-related AESIs by category for the fruquintinib plus BSC group were hypertension (30.5%), dermatological toxicity (28.3%), and thyroid dysfunction (19.1%) (Table 2). Grade ≥3 treatment-related AESI were reported in 24.1% of patients treated with fruquintinib plus BSC and 3.0% of patients who received placebo plus BSC. The most frequently reported grade ≥3 treatment-related AESIs by category for the fruquintinib plus BSC group were hypertension (11.2%), dermatological toxicities (6.4%), and liver function test abnormality (2.6%, for which 0.4% were increased AST and 0.2% were increased blood bilirubin) (Table 2). All grade and grade ≥3 treatment-emergent AESIs are reported in Supplementary Table S4. Considering treatment-related AESIs by preferred terms, the most frequently reported with fruquintinib plus BSC were hypertension (28.9%), PPE syndrome (18.6%), hypothyroidism (15.6%), and proteinuria (13.8%). The majority of these AESIs were low grade, with corresponding treatment-related grade ≥3 incidences of 10.7% for hypertension, 6.1% for PPE syndrome, and 1.5% for proteinuria. Treatment-related embolic and thrombotic events were only reported in 7 (1.5%) patients receiving fruquintinib plus BSC, although these were grade 3-4 in 6 of the 7 patients. Treatment-related gastrointestinal perforation events were reported in 6 patients (1.3%), all of which were grade ≥3; a further 10 patients had gastrointestinal perforation events that were not considered to be treatment-related giving an overall incidence of 3.5% with fruquintinib compared with 0.4% for placebo. Overall, 8 (1.8%) patients receiving fruquintinib plus BSC and 3 (1.3%) patients receiving placebo plus BSC had a treatment-emergent AESI leading to death. Of these, intestinal perforation in a patient who had a pre-existing history of low-volume peritoneal disease and multiple abdominal surgeries was the only treatment-related death reported with fruquintinib (<1%). There were no treatment-related deaths with placebo.

**Table 2.** Any-grade and grade ≥3 treatment-related AESI.

AESI category, *n* (%) PT	Fruquintinib + BSC (*n* = 456)		Placebo + BSC (*n* = 230)	
	Any grade	Grade ≥ 3	Any grade	Grade ≥ 3
*Hypertension* ^*^	139 (30.5)	51 (11.2)	12 (5.2)	2 (0.9)
Hypertension	132 (28.9)	49 (10.7)	12 (5.2)	2 (0.9)
*Dermatological toxicity*	129 (28.3)	29 (6.4)	17 (7.4)	0
PPE syndrome	85 (18.6)	28 (6.1)	6 (2.6)	0
*Hepatic function abnormal*	46 (10.1)	12 (2.6)	9 (3.9)	3 (1.3)
AST increased	24 (5.3)	2 (0.4)	2 (0.9)	1 (0.4)
ALT increased	22 (4.8)	5 (1.1)	1 (0.4)	0
Blood bilirubin increased	12 (2.6)	1 (0.2)	1 (0.4)	0
*Thyroid dysfunction*	87 (19.1)	2 (0.4)	3 (1.3)	0
Hypothyroidism	71 (15.6)	2 (0.4)	1 (0.4)	0
Blood thyroid stimulating hormone increased	18 (3.9)	0	2 (0.9)	0
*Infection*	11 (2.4)	2 (0.4)	2 (0.9)	0
*Proteinuria*	63 (13.8)	7 (1.5)	8 (3.5)	1 (0.4)
Proteinuria	63 (13.8)	7 (1.5)	8 (3.5)	1 (0.4)
*Hemorrhages*	33 (7.2)	3 (0.7)	5 (2.2)	0
*Embolic and thrombotic events*	7 (1.5)	6 (1.3)	1 (0.4)	1 (0.4)
*Gastrointestinal perforation*	6 (1.3)	6 (1.3)	0	0
*Left ventricular ejection fraction decreased*	3 (0.7)	2 (0.4)	3 (1.3)	0

^*^Includes hypertension, hypertensive crisis, increased diastolic blood pressure, increased blood pressure and hypertensive retinopathy.

Abbreviations: AESI, adverse event of specific interest; ALT, alanine aminotransferase; AST, aspartate aminotransferase; BSC, best supportive care; PPE, palmar-plantar erythrodysesthesia; PT, preferred term.

**Figure 1.**
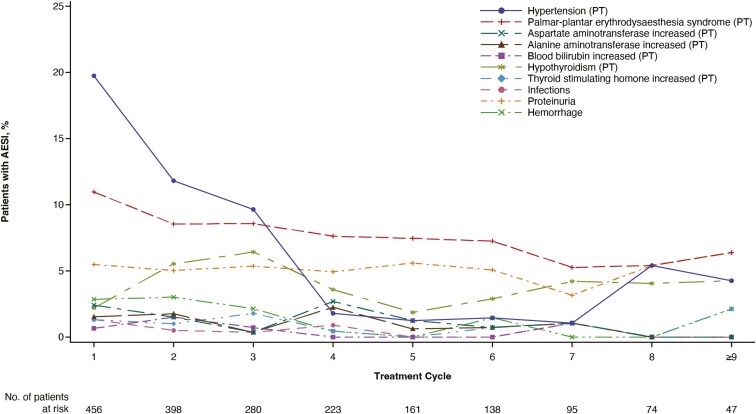
Occurrence of any grade treatment-related AESIs at each cycle with fruquintinib plus BSC (safety population). Abbreviations: AESI, adverse event of special interest; BSC, best supportive care; PT, preferred term.

Time to first occurrence of a treatment-related AESI with fruquintinib plus BSC is shown in **Table 3**. For patients receiving fruquintinib plus BSC, the time to first occurrence of a treatment-related AESI of any grade was typically within the first 3 cycles of treatment; hypertension and PPE had the earliest time to onset with a median time to first occurrence of 21 days. The proportion of treatment-related hypertension reported with fruquintinib was highest in cycle 1 at 19.7%, decreasing to 1.8% in cycle 4. The proportion of treatment-related PPE reported with fruquintinib was highest in cycle 1 (11.0%) and generally decreased with each cycle stabilizing at a rate of 6.4% in cycles 9 and beyond. The occurrence of the remaining treatment-related AESIs was generally consistent by treatment cycle (Figure 1). The pattern of occurrence of any grade treatment-emergent AESIs was similar to treatment-related AESIs and is shown in (Supplementary Figure S2). Treatment-related AESIs reported with fruquintinib plus BSC resolved rapidly (time from first onset to resolution); PPE syndrome had the longest time to resolution with a median of 39 days (range, 4-206), while hypertension was resolved in a median of 16.5 (range, 1-206) days (Table 4).

**Table 3.** Time to first occurrence of treatment-related AESIs.

Time (days) to first occurrence^*^	Fruquintinib + BSC (*n* = 456)	Placebo + BSC (*n* = 230)
Hypertension (PT), n	132	12
Median (Q1, Q3)	21.0 (5.0, 33.0)	20.0 (2.0, 29.5)
PPE syndrome/HFSR (PT), n	85	6
Median (Q1, Q3)	21.0 (12.0, 49.0)	13.0 (7.0, 24.0)
AST increased (PT), n	24	2
Median (Q1, Q3)	46.5 (21.0, 112.0)	99.5 (29.0, 170.0)
ALT increased (PT), n	22	1
Median (Q1, Q3)	49.0 (28.0, 112.0)	170.0 (170.0, 170.0)
Blood bilirubin increased (PT), n	12	1
Median (Q1, Q3)	49.5 (29.0, 58.5)	29.0 (29.0, 29.0)
Hypothyroidism (PT), n	71	1
Median (Q1, Q3)	58.0 (35.0, 113.0)	27.0 (27.0, 27.0)
Thyroid stimulating hormone increased (PT), n	18	2
Median (Q1, Q3)	50.0 (29.0, 77.0)	86.5 (60.0, 113.0)
Infection (AESI category), n	11	2
Median (Q1, Q3)	29.0 (20.0, 110.0)	39.0 (31.0, 47.0)
Proteinuria (AESI category), n	63	8
Median (Q1, Q3)	42.0 (29.0, 91.0)	29.0 (24.5, 44.0)
Hemorrhages (AESI category), n	33	5
Median (Q1, Q3)	42.0 (20.0, 61.0)	21.0 (19.0, 37.0)

^*^If a patient has multiple AEs occurrences under the same AESI category, the earliest AE onset data is as the first onset date to the AESI category.

Abbreviations: AESI, adverse event of special interest; ALT, alanine aminotransferase; AST, aspartate aminotransferase; BSC, best supportive care; HSFR, hand-foot skin reaction; PPE, palmar-plantar erythrodysesthesia; PT, preferred term; Q1, 25^th^ percentile; Q3, 75% percentile.

**Table 4.** Summary of resolution^*^ of treatment-related AESIs (safety population).

AESI^*^	Fruquintinib + BSC (*n *= 456)	Placebo + BSC (*n *= 230)
PPE syndrome (PT)		
Patients with event	85	6
Number of events resolved	79	4
Median time to resolution (range), days	39.0 (4-206)	31.5 (26-111)
Hypertension (PT)		
Patients with event	132	12
Number of events resolved	92	5
Median time to resolution (range), days	16.5 (1-206)	5.0 (3-81)
AST increased (PT)		
Patients with event	24	2
Number of events resolved	10	2
Median time to resolution (range), days	14.0 (5-77)	50.0 (15-85)
ALT increased (PT)		
Patients with event	22	1
Number of events resolved	10	2
Median time to resolution (range), days	14.0 (4-77)	50.0 (15-85)
Blood bilirubin increased (PT)		
Patients with event	12	1
Number of events resolved	5	0
Median time to resolution (range), days	6.0 (3-28)	-
Hypothyroidism (PT)		
Patients with event	71	1
Number of events resolved	9	1
Median time to resolution (range), days	30.0 (22-350)	36.0 (36-36)
Blood TSH increased (PT)		
Patients with event	18	2
Number of events resolved	7	1
Median time to resolution (range), days	38.0 (8-190)	85.0 (85-85)
Infections (AESI category)		
Patients with event	11	2
Number of events resolved	8	2
Median time to resolution (range), days	19.0 (9-71)	9.0 (5-13)
Proteinuria (AESI category)		
Patients with event	63	8
Number of events resolved	56	6
Median time to resolution (range), days	15.0 (2-191)	10.0 (3-91)
Hemorrhages (AESI category)		
Patients with event	33	5
Number of events resolved	33	3
Median time to resolution (range), days	8.0 (1-63)	1.0 (1-1)

^*^Resolution is defined as event status of resolved/recovered or resolved/recovered with sequalae.

Abbreviations: AESI, adverse event of special interest; ALT, alanine aminotransferase; AST, aspartate aminotransferase; BSC, best supportive care; PPE, palmar-plantar erythrodysesthesia; PT, preferred term; TSH, thyroid stimulating hormone.

In patients receiving fruquintinib plus BSC, 10.3% of patients required dose reductions, and 15.8% of patients required dose interruptions due to treatment-related AESIs, compared with 0.4% and 2.2%, respectively, for placebo plus BSC. The most common treatment-related AESIs resulting in dose reduction and dose interruption for fruquintinib were PPE syndrome (5.0% and 6.1%, respectively), hypertension (2.9% and 2.6%, respectively), and proteinuria (1.3% and 4.6%, respectively **Table 5**). The majority of dose reductions were to fruquintinib 4 mg with few patients requiring further reductions. Among patients receiving fruquintinib plus BSC only 5.9% discontinued due to any treatment-related AESI compared with 0.9% of patients receiving placebo plus BSC. The most frequent treatment-related AESI categories resulting in discontinuation of fruquintinib were embolic and thrombotic events (1.3%), gastrointestinal perforation (1.1%), and proteinuria (0.9%); the incidences of grade ≥3 treatment-related AESIs leading to discontinuation for individual preferred terms were all ≤1.3% (**Table 5**).

**Table 5.** Any-grade and grade ≥3 treatment-related AESIs leading to dose modification (reduction and interruption) and dose discontinuation (safety population)^*^.

AESI, *n* (%)	Leading to dose reduction				Leading to dose interruption				Leading to dose discontinuation			
	Fruquintinib + BSC (*n* = 456)		Placebo + BSC (*n* = 230)		Fruquintinib + BSC (*n* = 456)		Placebo + BSC (*n* = 230)		Fruquintinib + BSC (*n* = 456)		Placebo + BSC (*n* = 230)	
	Any grade	Grade ≥ 3	Any grade	Grade ≥ 3	Any grade	Grade ≥ 3	Any grade	Grade ≥ 3	Any grade	Grade ≥ 3	Any grade	Grade ≥ 3
Hypertension (PT)	13 (2.9)	13 (2.9)	1 (0.4)	1 (0.4)	12 (2.6)	11 (2.4)	1 (0.4)	0	2 (0.4)	1 (0.2)	0	0
PPE syndrome (PT)	23 (5.0)	14 (3.1)	0	0	28 (6.1)	14 (3.1)	0	0	3 (0.7)	2 (0.4)	0	0
AST increased (PT)	1 (0.2)	0	0	0	3 (0.7)	1 (0.2)	1 (0.4)	1 (0.4)	0	0	0	0
ALT increased (PT)	2 (0.4)	1 (0.2)	0	0	3 (0.7)	2 (0.4)	0	0	1 (0.2)	1 (0.2)	0	0
Blood Bilirubin increased (PT)	3 (0.7)	0	0	0	3 (0.7)	1 (0.2)	0	0	0	0	0	0
Hypothyroidism (PT)	0	0	0	0	1 (0.2)	1 (0.2)	0	0	0	0	0	0
Infection (AESI category)	1 (0.2)	0	0	0	3 (0.7)	2 (0.4)	0	0	0	0	0	0
Proteinuria (AESI category)	6 (1.3)	2 (0.4)	0	0	21 (4.6)	4 (0.9)	2 (0.9)	1 (0.4)	4 (0.9)	1 (0.2)	0	0
Hemorrhages (AESI category)	1 (0.2)	0	0	0	1 (0.2)	0	0	0	3 (0.7)	3 (0.7)	0	0
Embolic and thrombotic events (AESI category)	0	0	0	0	0	0	0	0	6 (1.3)	6 (1.3)	1 (0.4)	1 (0.4)
Gastrointestinal perforation (AESI category)	0	0	0	0	1 (0.2)	1 (0.2)	0	0	5 (1.1)	5 (1.1)	0	0
Left ventricular ejection fraction decreased (AESI category)	0	0	0	0	1 (0.2)	1 (0.2)	1 (0.4)	0	0	0	0	0

^*^No dose modifications were required for increased blood thyroid stimulating hormone.

Abbreviations: AESI, adverse event of specific interest; ALT, alanine aminotransferase; AST, aspartate aminotransferase; BSC, best supportive care; PPE, palmar-plantar erythrodysesthesia; PT, preferred term.

## Discussion

This safety analysis of FRESCO-2 demonstrates that fruquintinib plus BSC is well tolerated in pretreated patients with mCRC. As expected, the incidence of treatment-related AESIs observed upon receiving fruquintinib + BSC was higher than the incidence reported with placebo plus BSC (except decreased left ventricular ejection fraction, which was slightly higher with placebo plus BSC: 0.7% vs 1.3%). However, the majority of these were low-grade and manageable and did not result in treatment discontinuation. Placebo plus BSC had very few grade ≥3 treatment-related AESIs, therefore the percentage of most grade 1-2 treatment-related AESIs was incrementally increased with fruquintinib plus BSC compared with placebo plus BSC. Despite this, the median duration of treatment with fruquintinib plus BSC was almost twice as long as with placebo plus BSC, reinforcing the favorable therapeutic profile of fruquintinib. These data are encouraging, given FRESCO-2 was conducted in patients with pre-existing comorbidities and late-stage, pretreated disease (minimum of 3 prior lines), and a median age of 64.^20,25^

The most frequently reported (≥10%) all-grade treatment-related AESIs reported with fruquintinib plus BSC in FRESCO-2 were hypertension (28.9%), PPE syndrome (18.6%), hypothyroidism (15.6%), and proteinuria (13.8%). It is important to actively monitor and manage hypertension associated with VEGFR inhibition, including with fruquintinib. In FRESCO-2, 49.8% of patients in the fruquintinib plus BSC group and 52.2% of patients in the placebo plus BSC group reported hypertension as part of their medical history at enrollment. Despite these high incidences of hypertension as medical history, the incidences of grade ≥3 treatment-related hypertension reported in the study were 10.7% with fruquintinib plus BSC and 0.9% with placebo plus BSC. As most clinicians who treat patients with CRC are familiar with anti-VEGF therapy or VEGFR inhibition, in FRESCO-2 they were likely experienced in managing hypertension as a class effect.

Incidences of grade ≥3 PPE syndrome were reported in 6.1% of patients receiving fruquintinib plus BSC. The majority of cases of PPE syndrome were reversible (79/85; 92.9%) with only 3 cases leading to dose discontinuation, suggesting that this AE should not be prohibitive.

Reported incidences of hypothyroidism and proteinuria as medical history were low among patients in the fruquintinib plus BSC group (7.5% and 3.7%, respectively), as were grade ≥3 events in FRESCO-2 (0.4% and 1.5%, respectively). No cases of hypothyroidism and 0.9% of proteinuria cases resulted in dose discontinuations, and most cases of proteinuria (56/63; 88.9%) were reversible. Despite the majority of patients (74% in fruquintinib plus BSC and 67% in placebo plus BSC) having liver metastases, the incidence of treatment-related elevated transaminases and bilirubin was infrequent, and mainly low grade with fruquintinib: treatment-related grade ≥3 increased ALT was reported in 1.1% of patients, treatment-related grade ≥ 3 increased AST in 0.4% of patients, and treatment-related grade ≥3 increased blood bilirubin in 0.2% of patients.

The AESIs reported in FRESCO-2 were consistent with those reported with fruquintinib in the phase III FRESCO study conducted in China,^29^ and the incidences of the most frequently reported all-grade treatment-related AESIs in FRESCO were higher than in FRESCO-2. The most frequently reported treatment-related AESIs among patients in FRESCO were hypertension (all grade: 55.4%, grade 3-4: 21.2%), PPE syndrome (all grade: 49.3%, grade 3-4: 10.8%), and proteinuria (all grade: 42.1%, grade 3-4: 3.2%).^29^ In FRESCO, one grade 3 hepatic laboratory abnormality was reported in each treatment arm (fruquintinib and placebo), with no treatment-emergent grade 4 or 5 hepatic laboratory abnormalities observed.^24,29^ For both FRESCO and FRESCO-2, patients with severe hepatic impairment were excluded from enrollment, and therefore consistent monitoring of liver function is recommended during treatment with fruquintinib.^20,24,25,29^ In FRESCO-2, one patient who had a pre-existing history of low-volume peritoneal disease and multiple abdominal surgeries died due to an intestinal perforation after receiving fruquintinib, which was considered to be treatment-related by the investigator. Overall, 1.3% of patients receiving fruquintinib had a grade ≥ 3 treatment-related gastrointestinal perforation; however, in patients treated with fruquintinib in FRESCO there were no incidences of severe or fatal treatment-related gastrointestinal perforation. Although gastrointestinal perforation is rare, it is a potentially serious complication; patients should be periodically monitored and fruquintinib should be permanently discontinued in patients who develop a gastrointestinal perforation or fistula.^27,30^

The safety profile of fruquintinib was consistent with the understanding of the mechanism of action of VEGFR 1-3 inhibition, with hypertension, PPE syndrome, proteinuria, and hypothyroidism being previously reported effects of VEGFR, or multi-kinase inhibition.^29,31-34^ Patients were closely monitored in FRESCO-2 and AESIs were effectively managed with dose modifications and supportive treatments as described in the protocol. Improving selectivity in single-agent therapies may lead to reduced serious toxicities, such as hepatotoxicity observed with regorafenib, and myelosuppression observed with TAS-102.^16-19^ Of note in FRESCO-2, any grade all-cause or treatment-related neutropenia was reported in less than 5% of patients.^25^

In contrast to fruquintinib, which is a highly selective inhibitor of all 3 VEGFRs, regorafenib is a multi-kinase inhibitor, which in addition to VEGFR 1-3 targets several other protein kinases involved in angiogenesis, oncogenesis, and the tumor microenvironment.^23,35^ Regorafenib has been associated with rare but sometimes fatal hepatotoxicity,^18,19^ with a higher incidence of increased liver transaminases and bilirubin compared with placebo observed in a meta-analysis of 2213 patients from 14 trials,^36^ and one fatal regorafenib-induced liver injury reported in the primary phase III study.^13^ Consequently, patients receiving regorafenib need to be closely monitored with liver function tests obtained at least every 2 weeks during the first 2 months of treatment; in addition, regorafenib is not recommended for use in patients with severe hepatic impairment.^18,19^

PPE syndrome and hypertension have also been frequently reported with regorafenib in the primary placebo-controlled phase III trial.^13^ This was confirmed in a follow-up open-label phase IIIb single-arm trial of regorafenib in 2864 patients with treatment-refractory mCRC, with the incidence of treatment-related grade ≥3 PPE syndrome ranging from 14% to 17%.^37^ Although cross-trial comparisons of AE incidences should be avoided due to the differences in trial populations and assessments, the higher incidence of PPE syndrome observed with regorafenib versus fruquintinib is likely a result of dual inhibition of VEGFR and platelet-|derived epidermal growth factor receptor by regorafenib, both of which have been associated with the mechanism of PPE syndrome.^34^ The incidences of treatment-related hypertension observed with fruquintinib plus BSC in FRESCO-2 (any grade: 30.5%, grade ≥3: 11.2%) were slightly higher compared with regorafenib (treatment-related any grade: 28% and treatment-related grade ≥3: 7%),^13^ but these events were manageable and did not result in high incidences of discontinuation.

## Conclusion

In patients with refractory mCRC, fruquintinib plus BSC demonstrated a safety profile that was consistent with selective VEGFR inhibition with AEs that were primarily low grade and manageable resulting in low incidences of discontinuation. Fruquintinib is a novel oral treatment option for patients with mCRC, is well tolerated, and enriches the continuum of care for these patients by prolonging survival.

## Supplementary Material

oyae360_suppl_Supplementary_Tables_S1-S4_Figures_S1-S2

## Data Availability

The datasets, including the redacted study protocol, redacted statistical analysis plan, and individual participants’ data supporting the results reported in this article, will be made available from the completed study within 3 months from initial request, to researchers who provide a methodologically sound proposal. The data will be provided after its de-identification, in compliance with applicable privacy laws, data protection and requirements for consent and anonymization.
