# Supplementary material for: Fruquintinib versus placebo in patients with refractory metastatic colorectal cancer: safety analysis of FRESCO-2
Source: Oncologist. 2025 Mar 31;30(3):oyae360. doi: 10.1093/oncolo/oyae360 (PMC11957243; doi:10.1093/oncolo/oyae360)
Supplement: oyae360_suppl_Supplementary_Tables_S1-S4_Figures_S1-S2 [file oyae360_suppl_supplementary_tables_s1-s4_figures_s1-s2.docx]

**Supplementary Appendix**

**Supplementary Table S1.** Overview of dosing adjustments/treatment suggestions for hypertension, dermatological toxicity, abnormal liver function, proteinuria, decreased platelet count, and infection from the FRESCO-2 protocol.

| **AESI category**  Grading* | **Dose adjustment** | **Treatment suggestions** |
| --- | --- | --- |
| **Dermatological toxicity** | | |
| **Grade 1** | None. | Active supportive treatment can be adopted to relieve the symptoms; for example, moisturizing skin cream, lotion, or hydrophilic urea ointment can be used. |
| **Grade 2** | - Hold treatment. - If the AE recovers to grade 1 or baseline level within 14 days, resume treatment at the same dose level. | Active supportive treatment can be adopted to relieve the symptoms; for example, moisturizing skin cream, lotion, or hydrophilic urea ointment can be used. |
| **Grade 3** | - Hold treatment. - If the AE recovers to grade 1 or baseline level within 14 days, resume treatment with a dose reduction to the next lower dose level. | Active supportive treatment can be adopted to relieve the symptoms; should the same AE occur for 3 times or continues to occur after 2 times of dose reduction, the drug should be terminated. |
| **Grade 4** | Permanently discontinue study treatment. | Emergent medical treatment. |
| **Hypertension** | | |
| **Grade 1** | None. | Follow up as planned schedule. |
| **Grade 2** | None. | Treatment objective: lower the blood pressure to <140/90 mm Hg (or <130/80 mm Hg in patients with chronic renal disease and/or diabetes). |
| **Grade 3** | - If BP >160/100mmHg lasts for >7 days after initiation of antihypertensive treatment or modification of current antihypertensive treatment, treatment should be held. - If hypertension resolves to grade 1 or baseline level within 14 days, resume treatment with a dose reduction to the next lower dose level. | Treatment objective: lower the blood pressure to <140/90 mmHg (or <130/80 mm Hg in patients with chronic renal disease and/or diabetes). |
| **Grade 4** | Permanently discontinue study treatment. | Emergent medical treatment. |
| **Abnormal liver function** | | |
| **Grade 1** | None. | Follow up per planned schedule. |
| **Grade 2 or 3**  (Liver function is abnormal but the biochemical criteria for Hy’s Law^†^ are not met) | - Hold treatment. - If the AE recovers to grade 1 or baseline level within 14 days, resume treatment with a dose reduction to the next lower dose level. | Provide supportive care and increase the frequency of liver function monitoring to 1–2 times a week. |
| **Grade 2 or 3**  (Liver function is abnormal and the biochemical criteria for Hy’s Law^†^ are met) | The study drug should be terminated immediately. | Provide supportive care and increase the frequency of liver function monitoring to 2–3 times a week. Urgent medical intervention indicated. |
| **Grade 4** | The study drug should be terminated. | Urgent medical intervention indicated. |
| **Proteinuria^§^** | | |
| **Grade 1** Proteinuria 1+ by urinalysis; 24-hour urine protein quantification <1.0g | None. | Follow up at scheduled study visit |
| **Grade 2** Proteinuria 2+ by urinalysis; 24-hour urine protein quantification between 1.0g to <2.0g | None. | Provide supportive treatment and increase the frequency of urine monitoring to once a week; consult nephrologist if necessary. |
| **Grade 2**  Proteinuria 2+ by urinalysis; 24-hour urine protein quantification between 2.0g to <3.5g | - Hold treatment. - If the AE recovers to grade 1 or baseline level within 14 days, resume treatment with a dose reduction to the next lower dose level. | Provide supportive treatment and increase the frequency of urine monitoring to once a week; consult nephrologist if necessary |
| **Grade 3**  24-hour urine protein quantification ≥3.5g | - Hold treatment. - If the AE recovers to grade 1 or baseline level within 14 days, resume treatment with a dose reduction to the next lower dose level. | Provide supportive treatment and increase the frequency of urine monitoring to once a week; consult nephrologist if necessary  Should the same AE occur for 3 times or continue to occur after 2 times of dose reduction, the drug should be terminated. |
| **Decreased platelet count** | | |
| **Grade 1** | None. | Perform follow up visit as scheduled. |
| **Grade 2** | - Hold treatment. - If the AE recovers to grade 1 or baseline level within 7 days, resume treatment at the same dose level | Hematology test should be monitored every 2–3 days; active treatment for platelet elevation is recommended. |
|  | - Hold treatment. - If the AE recovers to grade 1 or baseline level within 14 days, resume treatment with a dose reduction to the next lower dose level. | Hematology test should be monitored every 2–3 days; active treatment for platelet elevation is recommended. |
| **Grade 3** | - Hold treatment. - If the AE recovers to grade 1 or baseline level within 14 days, resume treatment with a dose reduction to the next lower dose level. | Hematology test should be monitored every 2–3 days; active treatment (platelet transfusion) to elevate platelet count is recommended.  Hematology examination should be performed once every week in the follow up visit. |
| **Grade 4** | - Permanently discontinue study treatment. | Hematology test should be performed once daily until the AE recovers to grade 2 or a lower grade; platelet transfusion or other active treatment should be provided |
| **Hemorrhage** | | |
| **Grade 1** | None. | Perform follow up visit as scheduled. |
| **Grade 2** | - Hold treatment. If the AE recovers to grade 1 or baseline level within 14 days, resume treatment with a dose reduction to the next lower dose level. | Provide active treatment. |
| **Grade 3 or above^¶^** | Permanently discontinue study treatment. | Emergent medical intervention. |

*For abnormal liver function, this included increase of ALT, AST, and total bilirubin, whether or not the biochemical criteria for Hy’s Law had been met.

^†^Hy’s Law is an increase in serum AST or ALT ≥3 × ULN together with total bilirubin ≥2 × ULN, and no other reason can be found to explain the biochemical changes, for example, new or worsening hepatobiliary metastases, elevated serum alkaline phosphatase indicating cholestasis, viral hepatitis, another suspect drug, or any other specific cause of severe hepatocellular injury. The elevation in transaminases must precede or be coincident with (i.e., on the same day as) the elevation in total bilirubin, but there is no specified timeframe within which the elevations in transaminases and total bilirubin must occur.

^§^If protein ≥2+ on urinalysis during the study, a 24-hour urine test had to be conducted within 1 week, with dose modification following the result of 24-hour urine protein quantification.

¶Complete medical history, laboratory tests (hematology and biochemistry) and appropriate auxiliary examination of the patient were obtained by the investigator.

Abbreviations: AE, adverse event; AESI, adverse event of special interest; ALT, alanine aminotransferase; AST, aspartate aminotransferase; BP, blood pressure; ULN, upper limit of normal.

**Supplementary Table S2**. Relevant medical history reported at study entry (safety population).

| **PT (unless stated), n (%)** | **Fruquintinib + BSC (*n* = 456)** | **Placebo + BSC (*n* = 230)** |
| --- | --- | --- |
| **Hypertension** | 227 (49.8) | 120 (52.2) |
| **Hypothyroidism** | 34 (7.5) | 18 (7.8) |
| **Proteinuria** | 17 (3.7) | 4 (1.7) |
| **PPE syndrome** | 9 (2.0) | 3 (1.3) |
| **Infections and infestations SOC*** | 65 (14.3) | 38 (16.5) |
| **Investigations SOC^†^** | 46 (10.1) | 24 (10.4) |

*Includes PT urinary tract infection.

^†^Includes PTs AST increased, ALT increased, and blood bilirubin increase.

Abbreviations: ALT, alanine aminotransferase; AST, aspartate aminotransferase; BSC, best supportive care; PPE, palmar-plantar erythrodysesthesia; PT, preferred term; SOC, System Organ Class.

**Supplementary Table S3.** Most common (≥5%) any grade and grade ≥3 treatment-related AEs (safety population).

| **PT, n (%)** | **Fruquintinib + BSC (*n* = 456)** | | **Placebo + BSC (*n* = 230)** | |
| --- | --- | --- | --- | --- |
|  | Any grade | Grade ≥3 | Any grade | Grade ≥3 |
| Hypertension | 132 (28.9) | 49 (10.7) | 12 (5.2) | 2 (0.9) |
| Asthenia | 112 (24.6) | 24 (5.3) | 34 (14.8) | 3 (1.3) |
| PPE syndrome | 85 (18.6) | 28 (6.1) | 6 (2.6) | 0 |
| Diarrhea | 82 (18.0) | 15 (3.3) | 14 (6.1) | 0 |
| Decreased appetite | 73 (16.0) | 6 (1.3) | 18 (7.8) | 2 (0.9) |
| Hypothyroidism | 71 (15.6) | 2 (0.4) | 1 (0.4) | 0 |
| Fatigue | 63 (13.8) | 15 (3.3) | 21 (9.1) | 1 (0.4) |
| Proteinuria | 63 (13.8) | 7 (1.5) | 8 (3.5) | 1 (0.4) |
| Dysphonia | 63 (13.8) | 0 | 10 (4.3) | 0 |
| Stomatitis | 60 (13.2) | 7 (1.5) | 5 (2.2) | 0 |
| Mucosal inflammation | 58 (12.7) | 2 (0.4) | 6 (2.6) | 0 |
| Nausea | 43 (9.4) | 1 (0.2) | 19 (8.3) | 1 (0.4) |
| Vomiting | 38 (8.3) | 3 (0.7) | 11 (4.8) | 1 (0.4) |
| AST increased | 24 (5.3) | 2 (0.4) | 2 (0.9) | 1 (0.4) |

Abbreviations: AEs, adverse events; AST, aspartate aminotransferase; BSC, best supportive care; PPE, palmar-plantar erythrodysesthesia; PT, preferred term.

**Supplementary Table S4.** Any grade and grade ≥3 treatment-emergent AESIs (safety population).

| **AESI category, n (%)** PT | **Fruquintinib + BSC (*n* = 456)** | | **Placebo + BSC (*n* = 230)** | |
| --- | --- | --- | --- | --- |
|  | Any grade | Grade ≥3 | Any grade | Grade ≥3 |
| **Hypertension*** Hypertension | **175 (38.4)** 168 (36.8) | **64 (14.0)** 62 (13.6) | **20 (8.7)** 20 (8.7) | **2 (0.9)** 2 (0.9) |
| **Dermatological toxicity** PPE syndrome | **157 (34.4)** 88 (19.3) | **31 (6.8)** 29 (6.4) | **27 (11.7)** 6 (2.6) | **1 (0.4)** 0 |
| **Hepatic function abnormal** AST increased ALT increased Blood bilirubin increased | **113 (24.8)** 48 (10.5) 47 (10.3) 36 (7.9) | **38 (8.3)** 10 (2.2) 14 (3.1) 11 (2.4) | **44 (19.1)** 11 (4.8) 9 (3.9) 11 (4.8) | **21 (9.1)** 3 (1.3) 1 (0.4) 6 (2.6) |
| **Thyroid dysfunction** Hypothyroidism  Blood thyroid-stimulating hormone increased | **123 (27.0)** 94 (20.6) 32 (7.0) | **2 (0.4)** 2 (0.4) 0 | **4 (1.7)** 1 (0.4) 3 (1.3) | **0** 0 0 |
| **Infection** | **96 (21.1)** | **30 (6.6)** | **29 (12.6)** | **13 (5.7)** |
| **Proteinuria**  Proteinuria | **80 (17.5)**  79 (17.3) | **8 (1.8)**  8 (1.8) | **12 (5.2)**  12 (5.2) | **2 (0.9)**  2 (0.9) |
| **Hemorrhages** | **65 (14.3)** | **8 (1.8)** | **22 (9.6)** | **4 (1.7)** |
| **Embolic and thrombotic events** | **21 (4.6)** | **14 (3.1)** | **5 (2.2)** | **2 (0.9)** |
| **Gastrointestinal perforation** | **16 (3.5)** | **10 (2.2)** | **1 (0.4)** | **1 (0.4)** |
| **Left ventricular ejection fraction decreased** | **5 (1.1)** | **4 (0.9)** | **6 (2.6)** | **2 (0.9)** |

*Includes hypertension, hypertensive crisis, increased diastolic blood pressure, increased blood pressure, and hypertensive retinopathy.

Abbreviations: AESI, adverse events of special interest; ALT, alanine aminotransferase; AST, aspartate aminotransferase; BSC, best supportive care; PPE, palmar-plantar erythrodysesthesia; PT, preferred term.

**Supplementary Figure S1**. Median daily dose of fruquintinib by treatment cycle (safety population).

**
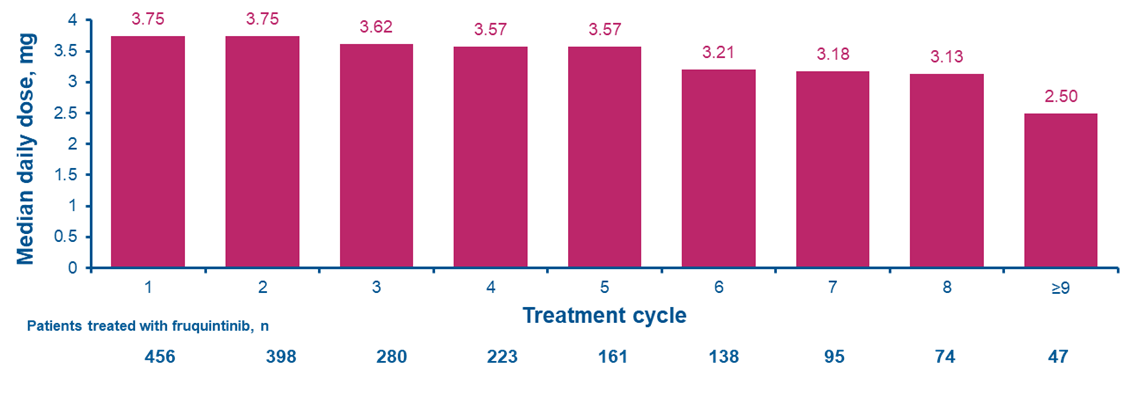
**

**Supplementary Figure S2**. Occurrence of any grade all-cause treatment-emergent AESIs by cycle with fruquintinib plus BSC (safety population).


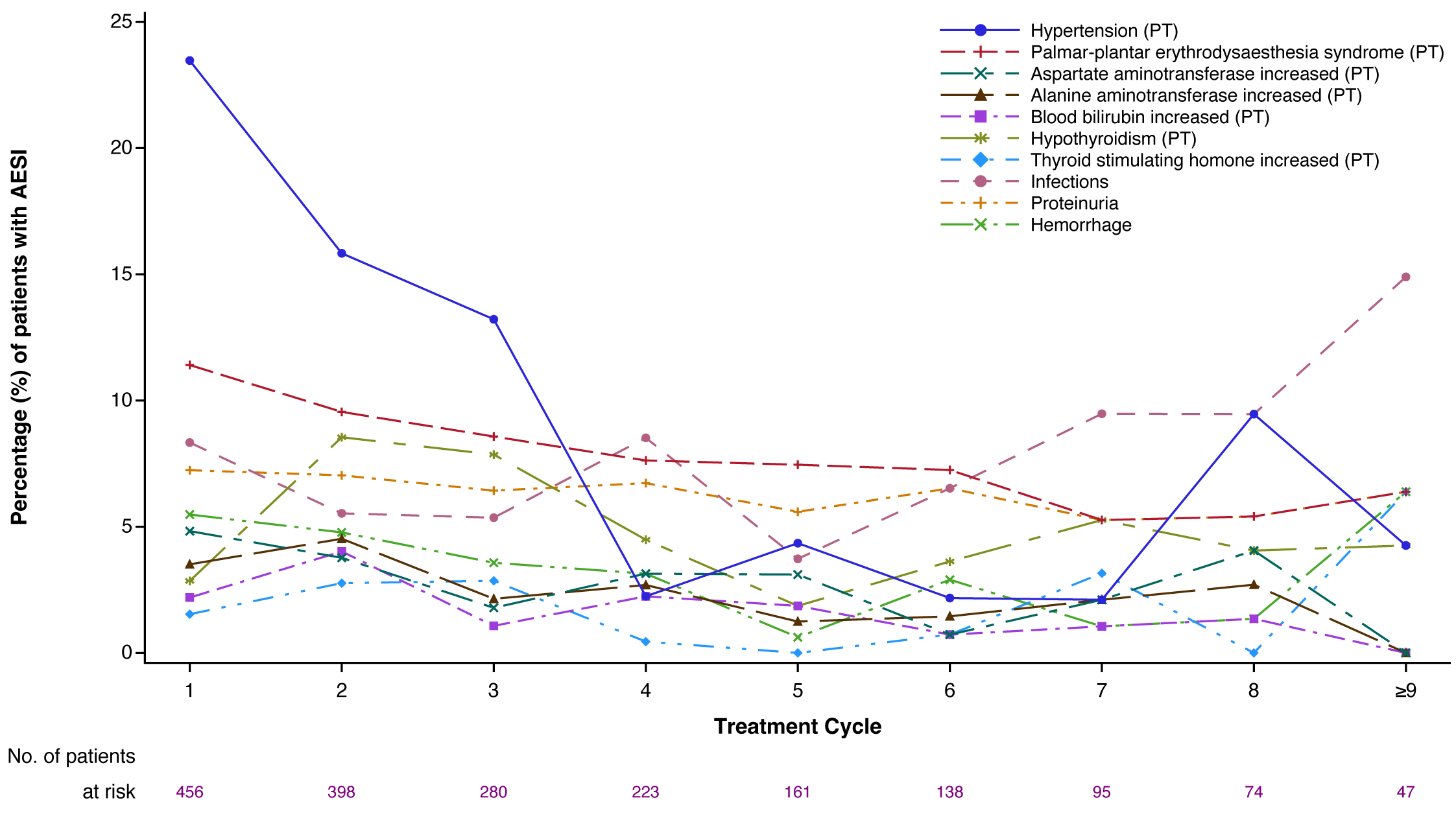


Abbreviations: AESI, adverse event of special interest; BSC, best supportive care; PT, preferred term.
